# L-Lactate Promotes Adult Hippocampal Neurogenesis

**DOI:** 10.3389/fnins.2019.00403

**Published:** 2019-05-24

**Authors:** Yaeli Lev-Vachnish, Sharon Cadury, Aviva Rotter-Maskowitz, Noa Feldman, Asael Roichman, Tomer Illouz, Alexander Varvak, Raneen Nicola, Ravit Madar, Eitan Okun

**Affiliations:** ^1^The Leslie and Susan Gonda Multidisciplinary Brain Research Center, Bar-Ilan University, Ramat Gan, Israel; ^2^The Mina and Everard Goodman Faculty of Life Sciences, Bar-Ilan University, Ramat Gan, Israel; ^3^The Paul Feder Laboratory on Alzheimer’s Disease Research, Ramat Gan, Israel

**Keywords:** L-lactate, neurogenesis, hippocampus, neuronal progenitor cells, MCT2

## Abstract

Neurogenesis, the formation of new neurons in the adult brain, is important for memory formation and extinction. One of the most studied external interventions that affect the rate of adult neurogenesis is physical exercise. Physical exercise promotes adult neurogenesis via several factors including brain-derived neurotrophic factor (BDNF) and vascular endothelial growth factor (VEGF). Here, we identified L-lactate, a physical exercise-induced metabolite, as a factor that promotes adult hippocampal neurogenesis. While prolonged exposure to L-lactate promoted neurogenesis, no beneficial effect was exerted on cognitive learning and memory. Systemic pharmacological blocking of monocarboxylate transporter 2 (MCT2), which transports L-lactate to the brain, prevented lactate-induced neurogenesis, while 3,5-dihydroxybenzoic acid (3,5-DHBA), an agonist for the lactate-receptor hydroxycarboxylic acid receptor 1 (HCAR1), did not affect adult neurogenesis. These data suggest that L-lactate partially mediates the effect of physical exercise on adult neurogenesis, but not cognition, in a MCT2-dependent manner.

## Introduction

Adult hippocampal neurogenesis, the process of forming new neurons in the adult brain, has sparked great interest following the observation that hippocampal granule cells are generated in adult rodents, a process which decreases significantly as the mice age (Kempermann, 2015). Whereas much is known about adult neurogenesis in rodents, it is still debatable whether and to what extent neurogenesis occurs in the adult human brain (Mathews et al., 2017; Boldrini et al., 2018; Sorrells et al., 2018). Multiple studies in rodents have shown that adult neurogenesis can modulate specific aspects of learning and memory (Sahay et al., 2011; Goncalves et al., 2016). Specifically, adult hippocampal neurogenesis is essential for certain memory processes. For example, reduced neurogenesis in the rat DG specifically affected the formation of memory trace in an eye blink conditioning task but not in a hippocampal-independent task (Shors et al., 2001). Moreover, enhancing survival of adult-born neurons improves performance in contextual fear-discrimination tasks (Sahay et al., 2011), which is an indication of improved pattern separation (Nakashiba et al., 2012), the ability to separate between similar stimuli, a DG-dependent process (Leutgeb et al., 2007). In addition, adult neurogenesis has been linked to cognitive flexibility, the ability to avoid interference between novel and previously formed memories, as was demonstrated in reversal learning of different tasks such as a Morris water maze (Epp et al., 2016), an active place avoiding task (Burghardt et al., 2012) and in a touch-screen discriminating task (Swan et al., 2014).

The most effective intervention to promote neurogenesis shown to date is physical exercise. Several studies have established that adult hippocampal neurogenesis is enhanced following voluntary (van Praag et al., 1999a,b, 2005) and forced (Ji et al., 2014; So et al., 2017) physical exercise, with concomitant beneficial effects on cognitive learning and memory (van Praag et al., 1999a; Barak et al., 2015). Nevertheless, our understanding of the mechanism(s) by which physical exercise affects adult neurogenesis and cognitive learning is incomplete.

Lactate is a metabolite released from muscle cells during and following intense physical exercise (Wahl et al., 2018). Its conversion from pyruvate by lactate dehydrogenase is facilitated under conditions of reduced oxygen availability (Marti et al., 1994; Valvona et al., 2016). As a result, following physical exercise, an acute increase in the levels of circulating blood lactate occurs, in a manner dependent on physical exercise intensity and individual cardiovascular fitness (van Hall, 2010; Hering et al., 2018). In addition to its role in replenishing pyruvate in the liver to provide substrates for gluconeogenesis, lactate is preferentially taken up and metabolized by neurons in an activity-dependent manner (Larrabee, 1995; Smith et al., 2003).

Previous studies showed that lactate accumulation in the blood during physical exercise and exogenous administration of L-lactate both increase the expression of vascular endothelial growth factor (VEGF)-A in the brain (Morland et al., 2017). Moreover, in traumatic brain injury and intracerebral hemorrhage, lactate accumulates around the hematoma to promote neurogenesis and angiogenesis, resulting in a neuroprotective effect (Alvarez et al., 2014; Zhou et al., 2018). Lactate has long been viewed as a waste product of glycolysis, but an increasing number of studies indicate that lactate functions not only as an energy source to cells in the brain but also as a signaling molecule (Mosienko et al., 2015; Zhou et al., 2018). Furthermore, it was shown that lactate supports synaptic activity (Schurr et al., 1988), long-term potentiation and long-term memory formation (Suzuki et al., 2011), presumably via transfer of lactate from astrocytes to neurons (Steinman et al., 2016; Magistretti and Allaman, 2018). In neurons, lactate enhances the expression of plasticity-related genes such as Arc, Zif268, and c-Fos through modulating NMDA receptor activity, thus increasing calcium influx and supporting neuronal activity (Yang et al., 2014).

Lactate enters the brain via several monocarboxylate transporters (MCTs) (Halestrap, 2013)—namely, MCT1 [Km(lactate) = 3.5 mM], mostly expressed on astrocytes and endothelial cells, MCT2 [Km(lactate) = 0.74 mM], mostly expressed on neurons, and MCT4 [Km(lactate) = 28 mM], which is expressed solely on astrocytes (Elizondo-Vega and García-Robles, 2017). In addition to lactate, these transporters also shuttle pyruvate and ketone bodies. However, lactate is the most important substrate shuttled via these transporters (Halestrap, 2013). In addition, hydroxycarboxylic acid receptor 1 (HCAR1) [previously known as G-protein coupled receptors (GPR)-81] mediates intracellular signaling following lactate-induced activation (Cai et al., 2008; Liu et al., 2009). HCAR1 is expressed by endothelial cells, adipocytes, neurons and to a lesser extent in astrocytes (Lauritzen et al., 2014). While HCAR1 activation in adipocytes leads to lipolysis (Liu et al., 2009), signaling via HCAR1 in endothelial cells promotes cerebral VEGF-A expression and angiogenesis (Morland et al., 2017).

As lactate transport via MCTs 1-4 and HCAR1 activation in the central nervous system (CNS) promotes neuronal activation and angiogenesis, we hypothesized that lactate accumulation in the blood at physical exercise-relevant rates and its influx to the brain enhances neurogenesis and hippocampus-dependent cognitive learning. To examine the specific effects of L-lactate on hippocampal neurogenesis and hippocampus-dependent cognitive learning, we injected mice with either L-lactate, an MCT2 antagonist (α-cyano-4-hydroxycinnamic acid, referred to hereafter as 4-CIN) or an HCAR1 agonist (3,5-dihydroxybenzoic acid, referred to hereafter as 3,5-DHBA). Our data indicates that L-lactate enhances adult hippocampal neurogenesis in a MCT2-, but not a HCAR1-dependent, manner. In contrast, we found no evidence for an effect of chronic L-lactate administration on cognitive learning in various hippocampus-dependent tasks.

## Materials and Methods

### Animals

Male C57bl/6 mice (Jackson Laboratories, Bar Harbor, ME, United States and Envigo, Jerusalem, Israel) were maintained under a reversed 12-h light/12-h dark cycle with food and water provided *ad libitum*. The animal numbers per group and ages are indicated where relevant for each experiment. In brief, for neurogenesis experiments we used 6-week-old mice (at the start of L-lactate injections), and for behavioral experiments we used 12-week-old mice (at the start of L-lactate injections). In all experiments, mice were housed in groups of four to five animals per cage. All procedures followed Bar-Ilan University’s guidelines and were approved by the Bar-Ilan University Animal Care and Use Committee.

### Drug Administration

Mice received daily intraperitoneal (i.p.) injections (200 μl) of either L-lactate solution (1.75 g/kg, Spectrum Chemicals, Gardena, CA, United States), 3,5-DHBA (270 mg/kg, D110000, Sigma, IL, United States), α-cyano-4-hydroxycinnamic acid (4-CIN, 90 mg/kg, C2020, sigma, IL) or phosphate buffered saline (PBS). When 4-CIN and L-lactate were co-administered, 4-CIN was administered initially, followed by L-lactate 1 h afterward. Injections of these compounds continued throughout all the experiments, including behavioral and metabolic analyses.

### Behavioral Paradigms

An assessment of cognitive behavior of the mice was conducted regarding the effects of L-lactate on adult hippocampal neurogenesis; this commenced when the mice were 19 weeks old and followed seven consecutive weeks of drug injections.

#### Elevated Zero Maze

Anxiety was assessed using the elevated zero maze (EZM), a 65 cm-high ring-shaped table, divided into four equal interchanging closed and opened sections. The ring was 7 cm wide and had an outer diameter of 60 cm. The closed sections were confined by 20 cm-high walls and a semi-transparent ceiling, whereas the opened sections had 0.5 cm high curbs at the edges to prevent the animals from falling. Illumination was kept at 1,300 lux and trial duration was 5 min. Total time spent (s) in the open section vs. the closed sections was recorded and compared between treatment groups. In this test and in all the behavioral procedures described below, animal activity was monitored using the ANY-maze automated video tracking system (Stoelting, IL, United States).

#### Open Field

Exploratory behavior in a novel environment was assessed using a 40 cm × 40 cm square open field arena with black walls and white floor. The outer 6.5 cm of the square was defined as the periphery, all four corners (6.5 cm × 6.5 cm rectangles) were defined as ‘Corners,’ and the inner 26.5 cm × 26.5 cm rectangle was defined as the center. Illumination was kept at 1,300 lux. Initially in the test, mice were placed in the center of the open field and were allowed to freely explore the arena for 5 min. Time spent in each of the zones (measured in seconds), average speed (measured in meters/second) and total distance traveled (measured in meters) were recorded and compared between treatment groups. No locomotor deficits were found in any of the groups according to speed measurements and the experimenter observation.

#### Radial Arm Water Maze

Spatial learning capacity was tested using the radial arm water maze (RAWM), constructed of a pool with a diameter of 150 cm, with eight 10 cm-wide, 60 cm-long transparent arms and an 8 cm × 8 cm platform located at the end of one arm. Water was kept opaque using white non-toxic paint, at a constant temperature of 26 ± 1°C. Room illumination was kept at ∼20 lux. To habituate the mice to the maze, on day 1, mice were given four trials of 60 s to find a visible platform. Animals that did not locate the target were gently guided to the platform by hand. All animals had 30 s of resting on the platform. Twenty-four hours following the habituation step, mice began the acquisition phase. The platform location was changed between the habituation and acquisition phases. In the acquisition phase, mice were required to search for a hidden platform located 1.5 cm under the water line. Four different visible extra-maze cues were present on the walls. Mice were placed in the central zone of the maze and were allowed 90 s to find the platform. This trial was repeated four times daily until no significant improvement in performance was identified. During the acquisition phase, the following parameters were recorded and compared between treatment groups: latency to reach platform, mean speed, total distance traveled, number of entries and total time in each arm.

#### Modified Barnes Maze

Long-term spatial learning was also assessed using a modified Barnes maze based on an apparatus utilized by Youn et al. (2012). The apparatus we utilized was constructed of a circular table with a diameter of 122 cm, containing 40 holes, each with a diameter of 5 cm, randomly placed at least 7 cm from each other and up to 5 cm from the perimeter of the table. This design was used to prevent the typical ‘serial search’ strategy typically seen in the regular Barnes maze. Illumination was measured at the center of the table and maintained at 1,300 lux in order to motivate the animals to search for the target hole. During the habituation phase, which lasted one day, each mouse was placed in a cylinder at the center of the maze. Five seconds later, the cylinder was removed, and the mouse was allowed to explore the environment for a single 120 s trial. Mice that found the target hole were able to enter the escape chamber, whereas mice that failed to do so were placed back in the cylinder, now located above the target hole. In the spatial acquisition phase, four extra-maze visual cues were presented on the walls surrounding the Barnes table. Mice were given 120 s per trial to find the target hole, for three trials per day, with no inter-trial interval. This procedure was repeated daily until no significant improvement in performance was identified. During the acquisition phase the following parameters were recorded and compared between treatment groups: latency to enter target hole, mean speed, total distance traveled and number of entries to each hole.

#### T-Maze

A variant of the T-maze alternation test modified from Deacon and Rawlins (2006) was used to test short-term hippocampus-dependent memory. Briefly, T-maze arms were 30 cm long and 15 cm wide, walls were 15 cm high, covered by black and white patterns. Mice were tested three times at 2 h intervals, and each test consisted of two stages. During acquisition, mice were released from the starting chamber and were given the opportunity to enter one of the target arms. Trials ended when the animals spent more than 2 s with all four limbs inside one of the target arms. The tracking software saved the chosen arm as ‘L’ (left) or ‘R’ (right). Next, mice were allowed to stay in the chosen arm for 30 s. The alternation rate was calculated in the repetitive trial, where mice were allowed to choose again between ‘L’ or ‘R.’

#### Spontaneous Alternation Test

The spontaneous alternation Y-maze apparatus consisted of three opaque white 40 cm long, 8 cm wide and 15 cm high arms at a 120° angle from each other. Mice were placed at the center of the Y-maze and were allowed to freely explore the maze for 5 min. The number of arm entries and their sequences were recorded in order to calculate the percentage and type of alternation. Spontaneous alternation performance (SAP) was assessed by scoring the pattern of entries into each arm during the 5 min of the test. Successive alternations were defined as entries into each of the three arms as on overlapping triplet sets (i.e., ABC, BCA). SAP percentage was defined as the ratio of actual (= total alternations) to possible (= total arm entries - 2) number of alternations × 100. The alternate arm returns (AARs) and same arm returns (SARs) were also calculated.

#### Grip Strength Test

Muscular strength was assessed using a grip strength measurement apparatus (grip strength meter, Ugo Basile, Italy). Each mouse was allowed to maintain grip on a wire mesh screen with its forelimbs. The tail was gently and steadily pulled to measure the maximum force until the mouse released the wire mesh screen. Grip strength was indicated as gram-force to release the wire mesh screen for each of the three trials. Grip strength was averaged from three consecutive trials.

#### Lactate Measurements

Blood-lactate concentrations were monitored using the Lactate Pro 2 device (Arkary, Japan). The tail snip method (Kim et al., 2018) was used to extract a blood drop which was placed on a lactate strip (Lactate Pro2 test strip) to receive an immediate indication of the concentration of lactate in the blood. Lactate measurements were conducted using a cohort of mice that was not exposed to behavioral tests or subjected to neurogenesis analysis.

#### Metabolic Cages

Metabolic performance was studied using an automated indirect calorimetry system (TSE Systems GmbH, Germany). Mice were individually housed in home cages and were allowed to acclimate for 3 days. Food and water consumption as well as oxygen consumption (VO_2_), carbon dioxide production (VCO_2_) and respiratory exchange ratio (RER, ratio between VCO_2_ and VO_2_) were continuously monitored for 72 h, which included three complete light/dark cycles.

#### 5-Bromo-2′-Deoxyuridine Incorporation for the Assessment of Neurogenesis

5-Bromo-2′-deoxyuridine (BrdU; MP Biomedicals) was dissolved in PBS and sterile filtered using 0.2 μm syringe filters. All mice were injected with a BrdU dose of 100 mg/kg body weight per injection to label dividing cells. Three experimental groups of mice were used in this study to quantify hippocampal progenitor cells as well as young and adult neurons. Transiently amplifying neuronal progenitor cells typically exhibit a cell cycle in which ∼20–30% of the cells are present at S phase, during which the cells readily uptake nucleotides including BrdU. Moreover, newly formed neurons exhibit elevated rates of apoptosis at 4 days and 14 days into their proliferation (Sierra et al., 2010). Thus, in order to obtain an accurate snapshot of progenitor cells (Sox2^+^BrdU^+^ cells), 24 h following BrdU incorporation, we injected the mice three times with BrdU at 8 h intervals, and following an additional 8 h, we culled the mice and measured the number of NPCs that received BrdU within a 24 h window. For migrating neuroblasts (DCX^+^BrdU^+^ cells), we injected the mice six times with BrdU at 8 h intervals (48 h of exposure to BrdU) to account for the reduced number of cells that would be observed due to apoptosis, and we culled the mice 5 days later and measured the number of migrating young neurons. For young adult neurons (NeuN^+^BrdU^+^ cells), we conducted BrdU injections over a span of 5 days to account for reduced numbers of cells due to early and late neurogenesis-related apoptosis (Sierra et al., 2010). Injections were conducted at 12 h intervals, as 8 h intervals are technically difficult to conduct for long periods of time. The quantification was conducted at 4 weeks following BrdU injections. Following injections, mice were returned to their home cages. Specified intervals between BrdU pulses and animal culling were chosen according to known maturation marker expression timelines (Wojtowicz and Kee, 2006; Taupin, 2007).

#### Immunofluorescence

Mice were subjected to a common perfusion/fixation protocol (Gage et al., 2012). Briefly, mice were anesthetized using ketamine/xylazine (100 mg/kg, and 10 mg/kg, respectively) and perfused transcardially with cold 4% paraformaldehyde (PFA) in 0.1M PBS (Gage et al., 2012). Brains were removed and postfixed in 4% PFA overnight and then sequentially cryoprotected in 20% and 30% sucrose in 0.1M PBS. Brains were then sectioned into 40 μm-thick slices on a freezing microtome in the coronal plane. All immunohistochemistry was completed as free-floating sections and mounted on gelatin-coated slides for analysis. For BrdU staining, sections were first immersed in a 2N HCl for 30 min at 37°C, followed by 0.1M borate buffer (pH 8.5) for 10 min at room temperature. Sections were then washed six times in 0.1% Triton X-100 in PBS for a total of 30 min. Non-specific binding was blocked with 20% normal horse serum and 0.1% Triton X-100 in PBS for 1 h. The primary antibodies used for staining were rat anti-BrdU (1:1000; Serotec, OBT0030), rabbit anti-SOX2 (1:1000; Abcam, ab97959), goat anti-doublecortin (DCX) (1:250; Santa Cruz, sc-8066) and mouse anti-NeuN (1:10,000; Millipore, MAB377). All antibodies were diluted in PBS supplemented with 0.1% Triton X-100 with 2% horse serum. Following 72 h primary antibody incubation, sections were washed three times in 0.1% Triton X-100 in PBS for a total of 15 min. Sections were subsequently incubated with a fluorescent-tagged secondary antibody (Alexa-488 or Alexa-568 1:1000; Invitrogen) and diluted in PBS supplemented with 0.1% Triton X-100 for 1 h at room temperature.

#### Confocal Microscopy

For confocal microscopy we used an inverted Leica DMi8 scanning confocal microscope, driven by LASX software (Leica Microsystems, Mannheim, Germany). The objective used was a CS2 20×/0.75 with XY pixel size of 284 nm squared. Excitation for GFP was done with a 488 nm laser and emission was detected between 495 and 534 nm and, for red, was done with a 552 nm laser and emission between 569 and 640 nm, with an average from four attempts. For large areas, multiple images were acquired, and tiles were automatically merged into a single image with the Leica software. High resolution images were acquired with a CS2 63×/1.40 oil objective at an oversampling of XY pixel of 44 nm squared, Z step of 223 nm, and pinhole at 0.9 AU, and submitted to automated internal Leica Lighting deconvolution software. Single-plane projection was carried out for the display.

#### Assessment of Neurogenesis Using Stereology

The hippocampus and DG were outlined based on an atlas of the mouse brain (Franklin and Paxinos, 2013). Quantification of stained cells was evaluated by stereological counts using the optical dissector method (West et al., 1991). Optical fractionator sampling was carried out on a Leica DM6000 microscope (Leica Microsystems) coupled to a controller module and a high-sensitivity 3CCD video camera system (MBF Biosciences, VT, United States), and an Intel Xeon workstation (Intel). Sampling was implemented using the Stereo Investigator software package (MBF Biosciences, VT, United States). Analyzed brain sections spanned from -1.22 to -3.4 mm from bregma point, with every fifth slice used for quantification. The first section for each brain was randomly selected in order to avoid a sampling location bias. Ten to twelve sections were used for quantification from each animal. After delineation of the SGZ, or the granular layer of the DG, at low magnification (10× magnification), the whole contour was imaged with 20–30 1 μ-thick Z-stack images using a 63 Å∼ oil immersion objective (N.A. 1.4). Acquired images were first processed with Huygens deconvolution software (Scientific Volume Imaging) to improve resolution and signal-to-noise ratio (26) and later processed offline using the optical dissector method. Additionally, only cells that were visibly co-labeled in *X*, *Y*, and *Z* axes with an antibody against BrdU+ and either Neun, DCX or Sox2, and not overlapping with adjacent cells, were counted. Cells were only counted if they did not intersect with the lines of exclusion on the counting grid in Stereo Investigator. As images were obtained as stacks, the experimenter surveyed the stack to ascertain for both co-labeling and avoid overlapping cells. The total number of the positive cell population was estimated in reference to the section volume and extrapolated for the total volume of the DG. The following parameters were set for cell counts: the counting frame was 140 μm × 104 μm × 15 μm (height Å∼ width Å∼ dissector height), the same size as the sampling grid for an exhaustive sampling regime of the hole contour, and a guard zone height of 2 μm was used. An experimenter blind to all treatment groups performed the stereological counts. The coefficient of error (CE) Gunderson (*m* = 1) values were between 0.04 and 0.08 for all animals (Gundersen et al., 1999).

#### Body Composition Analysis Using NMR

Lean and fat mass were measured using the Minispec LF90 nuclear magnetic resonance instrument (Bruker Optics, Billerica, MA, United States).

#### HPLC Analysis of 3,5-DHBA and 4-CIN in the Blood

Levels of 3,5-DHBA and 4-CIN in the blood were analyzed using a Hitachi Elite LaChrom HPLC system equipped with an autosampler, column oven and diode array detector. HPLC traces were acquired using EZChrom Elite v. 3.2.1 software. Extracted HPLC samples (10 μL) were injected onto a LiChroCART RP-18e column (125 mm × 4 mm ID, Merck, Germany). 4-CIN samples were eluted isocratically using a phosphate running buffer (25 mM Sodium Phosphate pH = 3.2) mixed with acetonitrile at a ratio of 9:1 phosphate buffer to acetonitrile. The flow rate was set to 1 ml/min, the column temperature was 30°C and elution time was set to 20 min. 4-CIN consistently eluted at 9.4 min, and the peak was monitored at the wavelengths of 235 nm and 325 nm. The latter wavelength was used for quantitation. Estimates of sample concentrations were calculated using a linear standard curved based on peak area integration of standard solutions of 1 μM, 10 μM, and 100 μM of 4-CIN. 3,5-DHBA samples were eluted using a gradient of phosphate running buffer (25 mM potassium phosphate pH = 3.2) as buffer A and methanol as buffer B. The gradient program was set up as follows: 0 min: 1% B, 3 min: 1% B, 17 min: 10% B, 18 min: 50% B, 19 min: 1%B. The flow rate was set to 1 ml/min, column temperature was 30°C, and elution time set to 25 min. 3,5-DHBA eluted at 11 min, and the peak was monitored at a wavelength of 295 nm. Estimates of sample concentrations were calculated using a linear standard curved based on peak area integration of standard solutions of 0.1 mM, 1 mM, 10 mM, and 50 mM of 3,5-DHBA.

### Statistical Analysis

One-way analysis of variance (ANOVA) was used to compare the effect of drug administration on adult neurogenesis. To evaluate the effect of L-lactate treatment on proliferation and differentiation of NPCs, unpaired two-tailed *t*-test was used. Two-way ANOVA was used to examine the effect of drug administration on memory performance. All statistical analyses were carried out using GraphPad Prism Software.

## Results

### Mimicking Physical Exercise-Induced Lactate Levels in the Blood

Physical exercise-induced circulating lactate levels depend on exercise intensity and the individuals’ lactate threshold. In C57bl/6 mice, So et al. (2017) drew a correlation between exercise intensity and L-lactate levels as measured at the end of either fatigue or mild exercise compared with non-running control mice. Immediately following fatigue or mild exercise, L-lactate levels reach ∼15 and ∼12 mM, respectively. Thus, in order to mimic mild-to-intensive physical exercise-induced lactate levels in the blood, mice were injected with 1.75 g/kg L-lactate (in PBS, *n* = 3), or PBS (*n* = 3) and lactate were measured in blood samples taken at multiple time points afterward. Lactate levels peaked to 15.2 ± 1.94 mM at 15 min following injection and decreased to baseline levels at 210 min following injection (Supplementary Figure S1A). This L-lactate dose (1.75 g/kg) was selected for injections as it mimics physical exercise-induced lactate levels in the serum of C57bl/6 mice (So et al., 2017).

### L-Lactate Promotes Adult Hippocampal Neurogenesis

**Figure 1:**
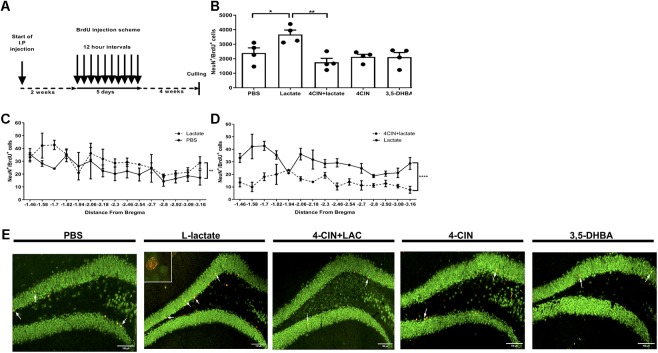
Exogenous L-lactate increases the number of NeuN^+^BrdU^+^ cells in the dentate gyrus. **(A)** Experiment design: Mice were i.p. injected with L-lactate, PBS, 4-CIN and 4-CIN, followed by L-lactate or 3,5-DHBA (*n* = 4 per treatment group), for 2 weeks prior to receiving 10 consecutive BrdU injections at 12 h intervals and sacrificed 4 weeks after the last injection. **(B)** Number of NeuN^+^/BrdU^+^ in the DG: NeuN^+^/BrdU^+^ cells were counted in the DG and the total DG population estimation was extrapolated for each animal. Results were averaged across treatment group. Number of NeuN^+^/BrdU^+^ cells in L-lactate-treated mice was significantly higher compared to PBS (^∗^*P* < 0.05) and 4-CIN + L-lactate (^∗∗^*P* < 0.01). **(C)** NeuN^+^/BrdU^+^ averaged cell distribution according to the distance from Bregma position. NeuN^+^/BrdU^+^ cell distribution was significantly higher in the L-lactate group compared with PBS treatment (^∗∗^*p* < 0.01) or **(D)** 4-CIN + L-lactate (^∗∗∗∗^*p* < 0.0001). **(E)** Representative NeuN^+^/BrdU^+^ cells in the SGZ of PBS, L-lactate, 4-CIN + L-lactate, 4-CIN, and 3,5-DHBA injected mice.

To assess the effects of L-lactate on adult hippocampal neurogenesis, we conducted daily injections to mice with either L-lactate, PBS, 4-CIN, sequential administration of 4-CIN and L-lactate, or 3,5-DHBA (*n* = 4 per treatment group). Two weeks into injections, mice were administered with 10 consecutive BrdU injections at 12 h intervals to label dividing cells (Figure 1A). Drug administration continued for five additional weeks to allow newly formed neurons to mature, following which the mice were sacrificed. Unbiased stereology was then used to quantify the number of newly formed matured neurons by counting NeuN^+^BrdU^+^ cells (Supplementary Figure S2A). A significant treatment difference was observed between the groups [*F*_(3,14)_ = 5.72, *P* < 0.01, one-way ANOVA, Figure 1B]. Specifically, compared with PBS injections, prolonged L-lactate administration resulted in a higher total number of NeuN^+^BrdU^+^ cells in the DG (2393 ± 356 and 3673 ± 300.1, respectively, *P* < 0.05, Figure 1B,E). A *post hoc* power analysis using G-power software (Faul et al., 2007) revealed that for one-way ANOVA analysis the power is 0.95 with an alpha of 0.05, effect size of 1.3 (as calculated from partial eta estimation using G-power software), and sample size of four animals per group. The effect size that was found in this study is considered to be large using Cohen’s (Cohen, 1988) criteria, and as a result the power is high. Therefore, we conclude that the sample size used in the study is sufficient to detect significant effect. It is well established that there are differences in the functionality and connectivity between the ventral and dorsal hippocampus (Sahay and Hen, 2007; Huckleberry et al., 2018). Also, differences along the dorsal-ventral axis are found in adult neurogenesis in the DG (Piatti et al., 2011). To investigate the distribution of the cells within the DG, the number of NeuN^+^BrdU^+^ cells was plotted according to the distance from bregma point. Higher numbers of NeuN^+^BrdU^+^ cells in L-lactate-treated mice were observed throughout the DG (*P* < 0.01, Figure 1C) without any concomitant effect on the volume of the DG (*P* > 0.05, Supplementary Figure S3).

### MCT2-Mediated L-Lactate Transport Mediates Adult Hippocampal Neurogenesis

L-Lactate enters neurons through MCT2, where it can function as a metabolite and/or as a signaling molecule (Bergersen, 2015; Mosienko et al., 2015). To assess whether lactate transfer to neurons via MCT2 mediates lactate-induced adult hippocampal neurogenesis, we first i.p. injected 4-CIN, an MCT2 antagonist (Poole et al., 1989) into mice (*n* = 4) in order to assess the kinetics of 4-CIN in the circulation. Ten minutes after injection, the levels of 4-CIN in the blood peaked rapidly to 20.3 μM and decreased to baseline levels 4 h afterward (Supplementary Figure S1B). The observed kinetics allowed us to assess whether pharmacologically antagonizing MCT2 abolishes the L-lactate-mediated effect on neurogenesis. Compared with L-lactate treated mice, mice receiving both 4-CIN and L-lactate exhibited significantly fewer NeuN^+^BrdU^+^ neurons (3673 ± 300.1 and 1755 ± 272.8, respectively, *P* < 0.05, Figure 1B,E), which was also evident throughout the DG axis (*P* < 0.01, Figure 1D). 4-CIN alone did not significantly affect the number of NeuN^+^BrdU^+^ neurons compared with PBS treatment (2128 ± 180.0 and 2393 ± 356, respectively, *P* > 0.05, Figure 1B). 4-CIN by itself or 4-CIN coupled with L-lactate did not result in an overall change in the volume of the dentate gyrus (*P* > 0.05, Supplementary Figure S3).

### L-Lactate-Induced Adult Hippocampal Neurogenesis Is Not Mediated by HCAR1

Since lactate can induce its effects via both metabolic pathways and via activation of HCAR1 (Barros, 2013; Bergersen, 2015; Morland et al., 2015), we assessed whether HCAR1 is involved in mediating L-lactate-induced effects on adult hippocampal neurogenesis. Similar to L-lactate, i.p. injection of 3,5-DHBA, a synthetic agonist of HCAR1 (Liu et al., 2012) into mice (*n* = 4) resulted in a rapid peak of 3,5-DHBA in the blood to a concentration of 1.75 mM at 30 min following injection, and decreased to baseline levels at 4 h following injection (Supplementary Figure S1C). No differences were observed in the number of NeuN^+^BrdU^+^ cells between 3,5-DHBA- and PBS-treated mice (2112 ± 315.1 and 2393 ± 356, respectively, *P* > 0.05, Figure 1B,E). 3,5-DHBA-treatment did not result in an overall change in the volume of the dentate gyrus (*P* > 0.05, Supplementary Figure S3). Despite the prolonged treatment with 3,5-DHBA, no effects were found on the animals’ body weight (*P* > 0.05, Supplementary Figure S4A), percentage of fat tissue (*P* > 0.05, Supplementary Figure S4B), percentage of lean tissue (*P* > 0.05, Supplementary Figure S4C) or absolute lean weight (*P* > 0.05, Supplementary Figure S4D) compared with PBS-treated mice. This was also evident in total cumulative food intake (*P* > 0.05, Supplementary Figure S5A) and total cumulative water intake (*P* > 0.05, Supplementary Figure S5B).

While a main effect for treatment was observed in the respiratory exchange rate (RER) [*F*_(1,14)_ = 4.9, *P* < 0.05, Supplementary Figure S5C], no significant differences were found in a *post hoc* multiple comparison analysis (*P* > 0.05); therefore, no conclusion can be drawn from the observed main effect in RER.

### NPC Proliferation or Early Differentiation Is Not Affected by L-Lactate

**Figure 2:**
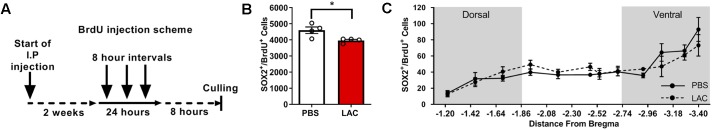
Exogenous L-lactate reduced the number of Sox2^+^BrdU^+^ cells in the dentate. **(A)** Experiment design: Mice were injected for 2 weeks with L-lactate (1.75 g/kg, *n* = 4) or PBS (*n* = 4) and were then injected with BrdU (100 mg/kg) three times at 8 h intervals and sacrificed 8 h after the last injection. **(B)** Number of Sox2^+^/BrdU^+^ in the DG; Sox2^+^/BrdU^+^ cells were counted for each experimental animal’s DG and total DG population estimation was extrapolated. Results were averaged across treatment group. L-lactate treatment reduces the number of Sox2^+^/BrdU^+^ cells (^∗^*p* < 0.05). **(C)** Sox2^+^/BrdU^+^ average cell distribution according to the distance from Bregma position. No significant differences were found between L-lactate and PBS throughout the DG (*P* > 0.05).

**Figure 3:**
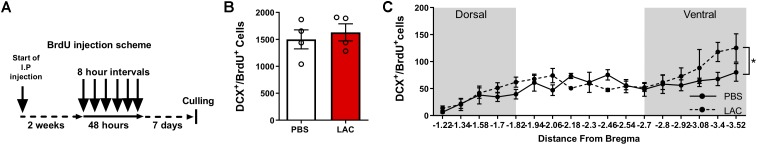
Exogenous L-lactate does not affect the number of DCX^+^BrdU^+^ cells in the dentate gyrus. **(A)** Experiment design: Mice were i.p. injected with 1.75 g/kg L-lactate solution (*n* = 4) or PBS (*n* = 4) and were then injected with BrdU for 2 days at 8 h intervals and sacrificed 5 days after the last injection. **(B)** Total number of DCX^+^/BrdU^+^ cells in the DG; DCX^+^/BrdU^+^ cells were counted for each experimental animal’s DG and total DG population estimation was extrapolated. Results were averaged across treatment group. No significant differences were found between L-lactate and PBS throughout the DG (*P* > 0.05). **(C)** DCX^+^/BrdU^+^ averaged cell distribution according to the distance from Bregma position. DCX^+^/BrdU^+^ cell distribution was significantly higher in L-lactate group compared with PBS treatment (^∗^*p* < 0.01).

To test whether L-lactate affects the early stages of adult neurogenesis, we quantified the pool of self-renewing cells by counting Sox2^+^BrdU^+^ cells (Supplementary Figure S2B) and the number of migrating neuroblasts by counting DCX^+^BrdU^+^ cells (Supplementary Figure S2C). To quantify Sox2^+^BrdU^+^ cells, we performed daily i.p. injections of L-lactate or PBS to mice (*n* = 4) for 2 weeks prior to injecting BrdU 3 consecutive times at 8 h intervals (Figure 2A). L-lactate treatment caused a decrease in the pool of Sox2^+^BrdU^+^ cells (3963 ± 70.4) compared with PBS treatment (4599 ± 203.5) (*P* < 0.05, Figure 2B). While a significant effect was found between the two groups, no such effect was observed throughout the DG (*P* > 0.05, Figure 2C). Therefore, we conducted a *post hoc* power analysis using G-power software, which indicated that the power is 0.69, whereas a power of 0.8 or above is needed for confidence in the data. To quantify DCX^+^BrdU^+^ cells, we performed daily i.p. injections of L-lactate or PBS into mice (*n* = 4) for 2 weeks prior to six consecutive injections of BrdU at 8 h intervals (Figure 3A). The total number of DCX^+^BrdU^+^ cells did not significantly differ between the L-lactate and PBS group (*P* > 0.05, Figure 3B). To investigate the bregma distribution along the DG, a two-way ANOVA was conducted. While a main effect for treatment was observed [*F*_(1,79)_ = 4.28, *P* < 0.05, Figure 3C], no significant differences were found in a *post hoc* multiple comparison analysis (*P* > 0.05); therefore, no conclusion can be drawn from the observed main effect throughout the DG.

### Chronic L-Lactate Administration Does Not Affect Hippocampal-Dependent Spatial Learning

**Figure 4:**
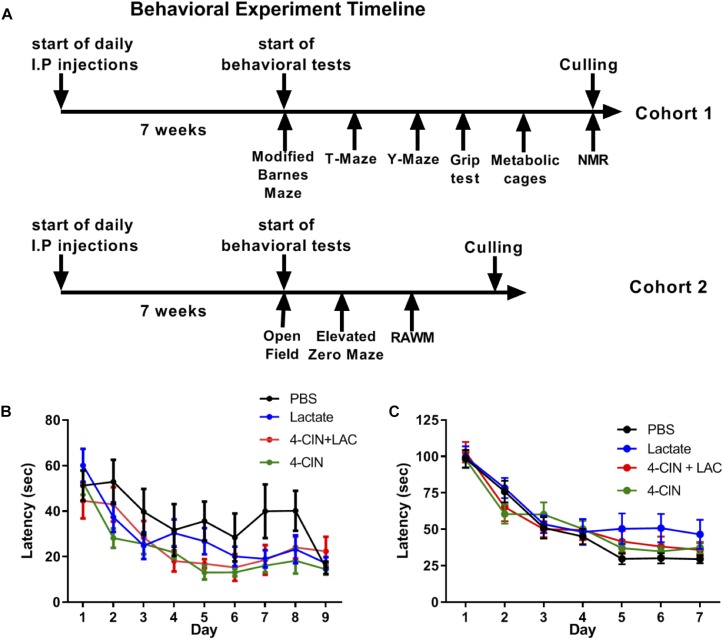
Chronic L-lactate administration does not affect long-term hippocampal-dependent spatial learning. **(A)** Experiment design: Mice were i.p. injected with L-lactate, PBS, 4-CIN and 4-CIN followed by L-lactate (*n* = 8–12 per group) for 7 weeks before starting behavioral experiments for nine additional weeks. During the behavioral experiments, treatments were continuously administered. Cohort 1 (upper timeline) was tested in the Barnes maze, T-maze, Y-maze, grip strength test, metabolic cages and finally underwent NMR analysis for body composition. Cohort 2 was tested in the open field arena, the elevated zero maze and the radial arm water maze. **(B)** Mice were tested using the RAWM; latency to reach platform. No significant effect was found between treatments (*P* > 0.05). **(C)** Mice were tested using the modified Barnes maze; latency to reach target hole. No significant effect was found between treatments (*P* > 0.05).

Lactate entry into neurons is essential for memory consolidation and immediate early gene expression through NMDA receptor sensitization (Yang et al., 2014). Moreover, the beneficial effects of physical exercise, a strong lactate inducer (Goodwin et al., 2007), on neurogenesis and hippocampal-dependent memory are well established (Baptista and Andrade, 2018). We thus hypothesized that lactate may be the metabolite which mediates the beneficial effects of physical exercise on long-term hippocampal-dependent memory formation through its effect on adult neurogenesis. To assess the impact of lactate on hippocampal-dependent cognitive learning and memory, mice received daily injections of either L-lactate (*n* = 11), PBS (*n* = 9), 4-CIN (*n* = 10) or 4-CIN followed by L-lactate (*n* = 9) for 7 weeks prior to initiating a battery of behavioral tests, during which treatments were continuously administered (Figure 4A). Despite these prolonged treatments, no differences were found between the different groups in animals’ body weight (*P* > 0.05, Supplementary Figure S4A), percentage of fat tissue (*P* > 0.05, Supplementary Figure S4B), percentage of lean tissue (*P* > 0.05, Supplementary Figure S4C) or absolute lean weight (*P* > 0.05, Supplementary Figure S4D). Thus, no gross differences in body composition could affect the subsequent behavioral aspects of the mice. This was also evident in total cumulative food intake (*P* > 0.05, Supplementary Figure S5D), total cumulative water intake (*P* > 0.05, Supplementary Figure S5E) and RER (*P* > 0.05, Supplementary Figure S5F).

To measure a possible effect of lactate on long-term spatial learning, we first used the RAWM (Buresova et al., 1985). To this end, mice were tested for 9 days, with three daily trials with no inter-trial interval, until mice no longer showed improvement in latency to reach the platform. No significant differences were found between the groups in latency to reach the target platform [*F*_(3,35)_ = 2.516, *P* > 0.05, Figure 4B]. In agreement with this, no differences were observed in reference memory errors (reflecting long-term spatial memory deficits) (*P* > 0.05, Supplementary Figure S6A) or working memory errors (reflecting short-term spatial memory deficits) (*P* > 0.05, Supplementary Figure S6B).

The RAWM utilized in this study presents an 8-choice decision point for the mice when located in the central arena and is considered a relatively simple spatial task. To further increase the spatial demand from the mice, we employed a variant of the Barnes maze, modified form (Feng et al., 2017, see methods), exhibiting 40 randomly placed holes on a white surface with one hole serving as an escape box. To this end, we injected a new cohort of mice with PBS (*n* = 12), L-lactate (*n* = 8), 4-CIN (*n* = 12), and 4-CIN + L-lactate (*n* = 10). These mice were then tested using the modified Barnes maze task for 5 days, with three daily trials with no inter-trial interval, until mice no longer showed improvement in latency to reach the platform. Again, no differences in latency to reach the escape box were found between the different groups [*F*_(3,39)_ = 2.06, *P* > 0.05, Figure 4C]. Moreover, no effect was noted on reference memory errors (*P* > 0.05, Supplementary Figure S6C) or working memory errors (*P* > 0.05, Supplementary Figure S6D).

Non-spatial hippocampal-dependent memory was not altered as a result of L-lactate, 4-CIN, or the combined L-lactate/4-CIN treatments as in the SAA test, and no treatment exhibited higher alternation (*P* > 0.05, Supplementary Figure S6E). A non-spatial variant of the T-maze also failed to reveal any differences between the groups (*P* > 0.05, Supplementary Figure S6F).

To rule out the possibility of anxiety response in L-lactate-treated mice, mice were tested in the EZM. No difference between the experimental groups was observed in time spent in the closed and open arms (*P* > 0.05, Supplementary Figure S6G) of the EZM. When allowed to freely explore an open field arena, which measures exploratory behavior, all experimental groups performed similarly and exhibited a similar amount of time in the corners of the arena (*P* > 0.05, Supplementary Figure S6H) suggesting that lactate does not affect anxiety or exploratory behavior when experiencing a novel environment. Motor impairments were not evident as a result of the different treatments, as testing the mice in the grip strength test revealed no differences amongst the different treatments (*P* > 0.05, Supplementary Figure S6I).

## Discussion

Since the discovery by van Praag et al. (1999b) that voluntary physical exercise enhances hippocampal adult neurogenesis, many efforts have been made to dissect the mechanisms behind this phenomenon. Physical exercise exerts both rapid and gradual long-term effects on the brain. These rapid changes include neurotransmitter level alteration and blood flow changes, followed by growth factor upregulation, most notably brain-derived neurotrophic factor (BDNF) and VEGF. Long-term changes include effects on cell proliferation and the generation of new neurons in the hippocampus (Vivar and van Praag, 2017). During prolonged physical exercise, oxygen levels decrease in muscle tissues, promoting reduction of pyruvate into lactate, which accumulates and is released into the circulation (van Hall, 2010). Ample evidence indicates lactate as a pleiotropic molecule with increasing importance, as our understanding of its roles in health and disease expands. During physical exercise, there is an increase in blood lactate concentrations that leads to an influx of lactate into the brain (Ide et al., 2000; Quistorff et al., 2008). L-lactate is now accepted as an important metabolite in the CNS, especially under high energy demand (Mosienko et al., 2015). For this reason, we have investigated the possible roles of lactate in mediating the effects of physical exercise on neurogenesis and cognitive learning. Herein, we describe an important causative effect for lactate in promoting adult hippocampal neurogenesis.

Previous studies showed that physical exercise induces a twofold to threefold increase in adult neurogenesis (van Praag et al., 1999a,b). This is in contrast to our results, which showed a 1.5-fold increase in neurogenesis following L-lactate treatment. In addition to elevating lactate levels, physical exercise also affects numerous other processes, such as changes in oxygen supply, metabolic rate and the induction of growth factors such as BDNF and VEGF. These additional effects could account for the stronger impact exerted by physical exercise on neurogenesis levels compared with L-lactate treatment.

Our experimental design aimed at exposing mice to circulating lactate levels that are similar in concentration and kinetics to those exhibited during and after prolonged daily physical exercise. In addition to its peripheral effects, exogenously administered L-lactate was previously shown to effectively cross the blood–brain barrier (BBB), where it can exert central effects (Mächler et al., 2016; Carrard et al., 2018). Examining blood levels of lactate following L-lactate injection exhibited accumulations of lactate that resemble high-intensity physical exercise (Okamoto et al., 2015; Matsui et al., 2017; Matsukawa et al., 2017; So et al., 2017). High-intensity physical exercise is associated with lactate accumulation, hence an imbalance between lactate production and its removal (Ferreira et al., 2007). This imbalance in lactate is due to an increased rate of glycolysis that exceeds the rate of mitochondrial pyruvate utilization due to reduced oxygen availability. Exercise intensity greatly impacted the magnitude of the effects on hippocampal plasticity and consequentially cognitive behavior. It is thought that high-intensity stress triggers a stress response which rescues the beneficial effects of physical exercise. Nevertheless, the vast majority of high-intensity forced exercise studies employed a treadmill which includes a tail-shock to promote animals’ running. Thus, it is yet to be studied whether it is the actual intensity of the physical exercise or the stress involved in tail shocks that accounts for the blunted beneficial effect of high-intensity exercise (Triviño-Paredes et al., 2016).

Interestingly, the L-lactate-induced increase in neurogenesis was not due to increased cell proliferation or increased neuroblast differentiation, but rather an increase in the number of surviving newly born mature neurons, whereas physical exercise affects both NPC proliferation and differentiation as well as neuronal maturation (Ma et al., 2017). This effect may also depend on the type of physical exercise, intensity and individual cardiovascular fitness. Previous studies show that moderate physical exercise increases cell proliferation, differentiation and maturation, whereas a more intensive physical exercise only enhanced differentiation and maturation (Nokia et al., 2016; So et al., 2017). There are, however, some contrasting results in the literature regarding the effects of different physical exercise intensities on neurogenesis, neurotrophic factor release and memory capacity (Lou et al., 2008; Lezi et al., 2013; Afzalpour et al., 2015). While our study shows that the number of newly formed neurons increased, we cannot rule out the possibility that this increase is due to an increase in cell survival and/or decreased apoptosis rate during differentiation and maturation. Further research is needed to delineate these possibilities.

Circulating lactate can penetrate endothelial cells via MCT1 to enter neurons via MCT2 or to activate HCAR1 on neurons. 4-CIN is a competitive, non-transportable inhibitor of MCTs, capable of crossing the BBB (Schurr et al., 2001). The concentration used in our study inhibits MCT2 but not MCT1 and MCT4 (Erlichman et al., 2008). Antagonizing MCT-2 prior to administering lactate abolished L-lactate-induced neurogenesis. These results suggest that L-lactate mediates its effects on neurogenesis through activating MCT2 receptors on newly formed neurons. Mature neurons expressing MCT2 are also potential targets of L-lactate-induced metabolic and signaling effects which indirectly enhance adult neurogenesis. The effects of L-lactate through MCT2 may be mediated by modulating NMDA receptor activity and thereby enhancing the expression of plasticity genes, as previous studies have shown (Suzuki et al., 2011; Yang et al., 2014; Margineanu et al., 2018).

Surprisingly, HCAR1 activation by 3,5-DHBA did not mimic the effect of L-lactate on neuronal maturation, which may indicate that lactate exerts its effects on neurogenesis independently of HCAR1 activation. Alternatively, although 3,5-DHBA was shown to regulate VEGF levels in the CNS, it is possible that 3,5-DHBA cannot penetrate the BBB or activate HCAR1 in concentrations high enough to affect neurogenesis. While 3,5-DHBA was not previously directly shown to cross the BBB, other highly similar molecular structures, namely 2,5-DHBA and 2,3-DHBA, were shown to penetrate the BBB (Kostrzewa et al., 2000; Nowak et al., 2010). Despite the fact that 3,5-DHBA is a small molecule for which there is a high probability of crossing the BBB, there is a possibility that 3,5-DHBA does not adequately cross the BBB.

As lactate is involved in regulating neuronal excitability via the NMDA receptor (Yang et al., 2014; Jourdain et al., 2018), and since physical exercise improves various cognitive traits (van Praag et al., 2005), we hypothesized that lactate treatment exerts a beneficial effect on multiple cognitive behaviors in mice. Surprisingly, although prolonged L-lactate treatment elevated the number of newly formed neurons, it did not result in a beneficial effect in either short- or long-term spatial learning.

It is well established that physical exercise benefits both hippocampal-dependent cognitive learning as well as adult hippocampal neurogenesis (van Praag et al., 2005). Our results indicate that there is a possible dissociation between the effects of physical exercise-induced lactate on neurogenesis and cognitive learning. Although we cannot rule out a possible effect for lactate-induced neurogenesis on pattern separation, our data clearly show that lactate confers no beneficial effect on short- or long-term spatial learning. Moreover, a recently published study showed that enhancing adult neurogenesis alone is not sufficient to mimic physical exercise-induced memory improvement in an Alzheimer’s mouse model, while increasing BDNF expression and activation of adult neurogenesis improved memory performance (Choi et al., 2018). It is also possible that hippocampal neurogenesis affects other subtle cognitive traits such as the spatial strategies that are utilized by the mice (Gil-Mohapel et al., 2013). However, we are yet to develop an unbiased spatial strategy classifier for the modified Barnes maze similar to those we previously developed for the Morris water maze (Illouz et al., 2016b) and the classical Barnes maze (Illouz et al., 2016a). These recent results suggest that the beneficial effects of physical exercise on learning and memory may be mediated by mechanisms that are unrelated to L-lactate or neurogenesis.

To the best of our knowledge this is the first report on the effects of L-lactate on adult neurogenesis and the survival of newly formed neurons under normal physiological conditions. Previously published studies demonstrate the effect of L-lactate under extreme non-physiological conditions such as ischemia, traumatic brain injury and stroke. Our results indicate that L-lactate plays a physiological role during maturation of newly formed neurons in the dentate gyrus and may be involved in mediating both mild and intense physical exercise-induced neurogenesis.

## Ethics Statement

This study was carried out in accordance with the recommendations of the Bar-Ilan University Ethics committee. The protocol was approved by the institutional animal use committee.

## Author Contributions

YL-V, SC, AR-M, NF, and RM have conducted the experiments. TI has conducted bioinformatics and statistical analysis. EO, YL-V, RN, and RM have wrote the manuscript. AV conducted the HPLC experiments. AR has provided technical experimental help. EO has designed the experiments.

## Conflict of Interest Statement

The authors declare that the research was conducted in the absence of any commercial or financial relationships that could be construed as a potential conflict of interest.
