# Supplementary material for: L-Lactate Promotes Adult Hippocampal Neurogenesis
Source: Front Neurosci. 2019 May 24;13:403. doi: 10.3389/fnins.2019.00403 (PMC6542996; doi:10.3389/fnins.2019.00403)
Supplement: Supplementary file 1 [file Data_Sheet_1.docx]

**Supplementary Material**

**Figure S1. Levels of L-lactate, 4-CIN and 3,5-DHBA in blood after i.p injection.**Naïve mice were i.p injected with either L-lactate (n=8) or 4-CIN (n=3) or 3,5-DHBA (n=3).
Mice had access to food and water prior to injection. Blood concentrations were measured in several time points after the injection. **(A).** Blood-lactate concentrations were measured using the Lactate Pro 2. Lactate levels peaked to 15.2±1.94mM at 15min (P<0.0001) and decreased to baseline levels at 210min following 1.75gr/kg injection. **(B).** Blood-4-CIN concentrations were analyzed by HPLC. Levels of 4-CIN in the blood peaked rapidly at 20.3µM, 10min following injection, and decreased to baseline levels at 4h following 90mg/kg injection **(C).** Blood-3,5-DHBA concentrations were analyzed by HPLC. Levels of 3,5-DHBA peaked rapidly at 1.75mM 30min following injection and decreased to baseline levels at 4h following 270mg/kg injection.

**Figure S2. Validation of BrDU co-expression with the cellular identity markers NeuN, Sox2 and DCX.** Hippocampal slices were co-stained for BrdU and markers NeuN, Sox2 and DCX for analysis using confocal microscopy. **(A)** Left panels: BrDU^+^NeuN^+^ cells show co-expression in the cell nucleus. Right panel: expression histogram. **(B)** Left panel: BrDU^+^Sox2^+^ cells in the subgranular layer are indicated with white arrows. Middle panels: BrDU^+^Sox2^+^ cells show co-expression in the cell nucleus. Right panel: expression histogram. **(C)** Left panel: BrDU^+^Dcx2^+^ cells in the subgranular layer are indicated with white arrows. Middle panels: BrDU^+^Dcx^+^ cells show co-expression of BrDU in the nucleus of cells with cytoplasmic expression of Dcx. Right panel: expression histogram.

**Figure S3.** **L-lactate, 4-CIN or 3,5-DHBA do not affect DG volume**. DG volume of mice treated with L-lactate, PBS, 4-CIN, 4CIN followed by L-lactate, or 3,5-DHBA for the analysis of NeuN^+^/BrdU^+^. DG volume did not alter between the treatments (P>0.05).

**Figure S4. Body composition analysis.** Mice (PBS (n=8), L-lactate (n=8), 4-CIN (n=11), 4-CIN followed by L-lactate (n=13), 3,5-DHBA (n=11)) received daily i.p injections of L-lactate ,PBS, 4-CIN, 4-CIN followed by L-lactate or 3,5,-DHBA for 7 weeks. Mice were then subjected to body composition analysis by using nuclear magnetic resonance. No differences were found between the different groups in animals’ **(A)** body weight (gr) (P>0.05), **(B)** %Fat (P>0.05), **(C)** %Lean weight (P>0.05) or **(D)** lean weight (gr) (P>0.05).

**Figure S5. Prolonged L-lactate, 4-CIN or 3,5-DHBA treatments do not affect metabolic profile of the mice.** Metabolic performance was studied using automated indirect calorimetry system. Treated mice (L-lactate (n=3) or PBS (n=6) or 4-CIN (n=3) or 4-CIN+L-lactate (n=4) or 3,5-DHBA (n=4)) were individually housed and allowed to acclimate for 3 days in the metabolic cage. After acclimation, **(A, D)** food and **(B, E)** water consumption were measured, as well as **(C, F)** respiratory exchange ratio (RER, ratio between VCO2 and VO2). No difference was observed between the groups (P>0.5).

**Figure S6. Chronic L-lactate administration does not affect hippocampal-dependent spatial. (A)** Mice were tested in the RAWM**;** Reference memory errors in the RAWM. Numbers of reference memory errors were calculated for each day in the acquisition phase. Reference memory is recorded when a mouse visits an arm that is not the target arm. No significant effect was found between treatments (P>0.5). **(B)** Working memory errors in the RAWM. Number of working memory errors were calculated for each day of acquisition. Working memory error is recorded when a mouse enters a non-target arm more than once. No significant effect was found between treatments (P>0.5). **(C)** Mice were tested in the Modified Barnes maze; Reference memory errors. Numbers of reference memory errors were calculated for each day in the acquisition phase. Reference memory was recorded when a mouse visited a hole that is not the target hole. No significant effect was found between treatments (P>0.5). **(D)** Working memory errors. Working memory errors were recorded when a mouse entered a non-target hole more than one time. No significant effect was found between treatments (P>0.5). **(E)** Mice were tested in the Spontaneous alternation test; Short term memory was assessed using spontaneous alternation test. No significant effect was found between treatments (P>0.5) in the number of possible sequences SAP, AAR, SAR. **(F)** Mice were tested in the T-maze; Short term memory was assessed also by the T-maze test. No significant effect was found between treatments (P>0.5) in average success score. **(G)** Mice were tested in the Elevated Zero Maze; Anxiety was assessed using the elevated zero maze. Time spent in open/closed section did not differ between groups. **(H)** Mice were tested in the Open Field; Exploratory behavior in the open field arena. Left: time spent in the center, corners and periphery of the Open Field arena did not differ between groups. Right: heat maps of the Open Field test demonstrates the preferred time spent in the corners by all groups. **(I).** Mice were tested in the Grip strength test; Muscular strength was assessed using a grip strength measurement apparatus. Grip strength was indicated as gram force to release the wire mesh screen for each of the three trials. Grip strength was averaged from three consecutive trials. No significant effect was found between treatments (P>0.5).
