# Supplementary figures and images for: L-Lactate Promotes Adult Hippocampal Neurogenesis
Source: Front Neurosci. 2019 May 24;13:403. doi: 10.3389/fnins.2019.00403 (PMC6542996; doi:10.3389/fnins.2019.00403)

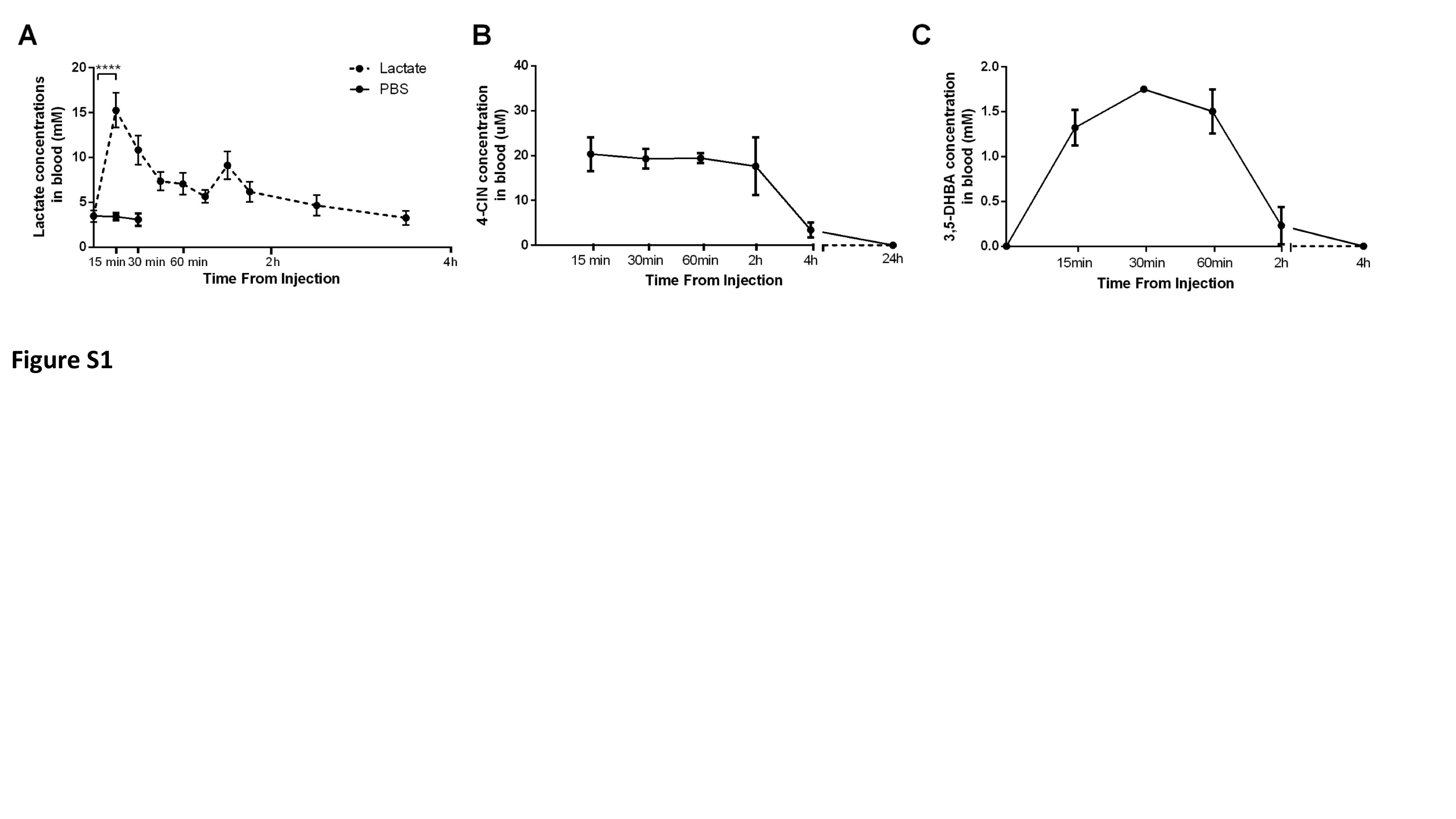

Supplement: Supplementary file 2 [file Image_1.TIFF]

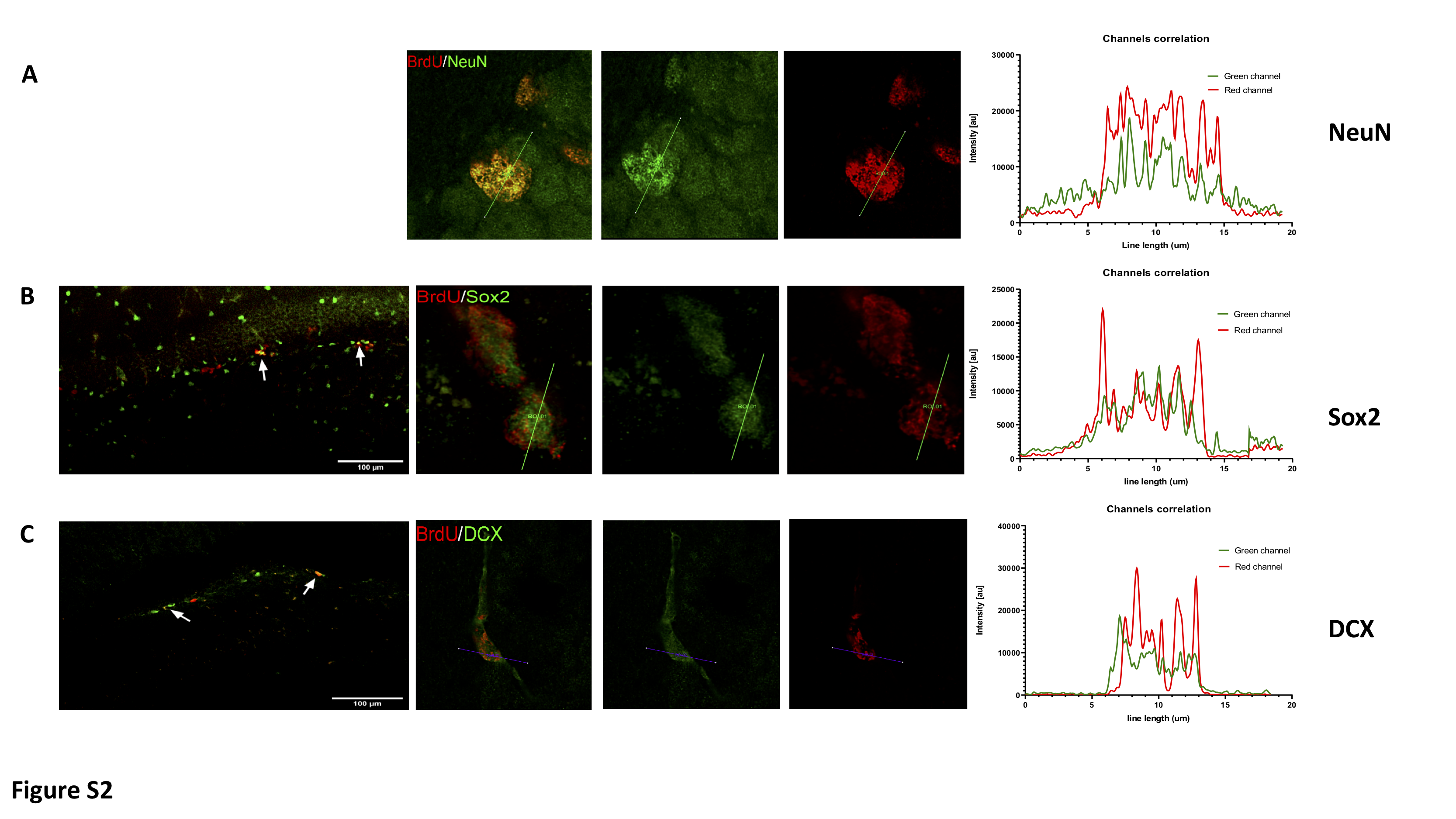

Supplement: Supplementary file 3 [file Image_2.TIFF]

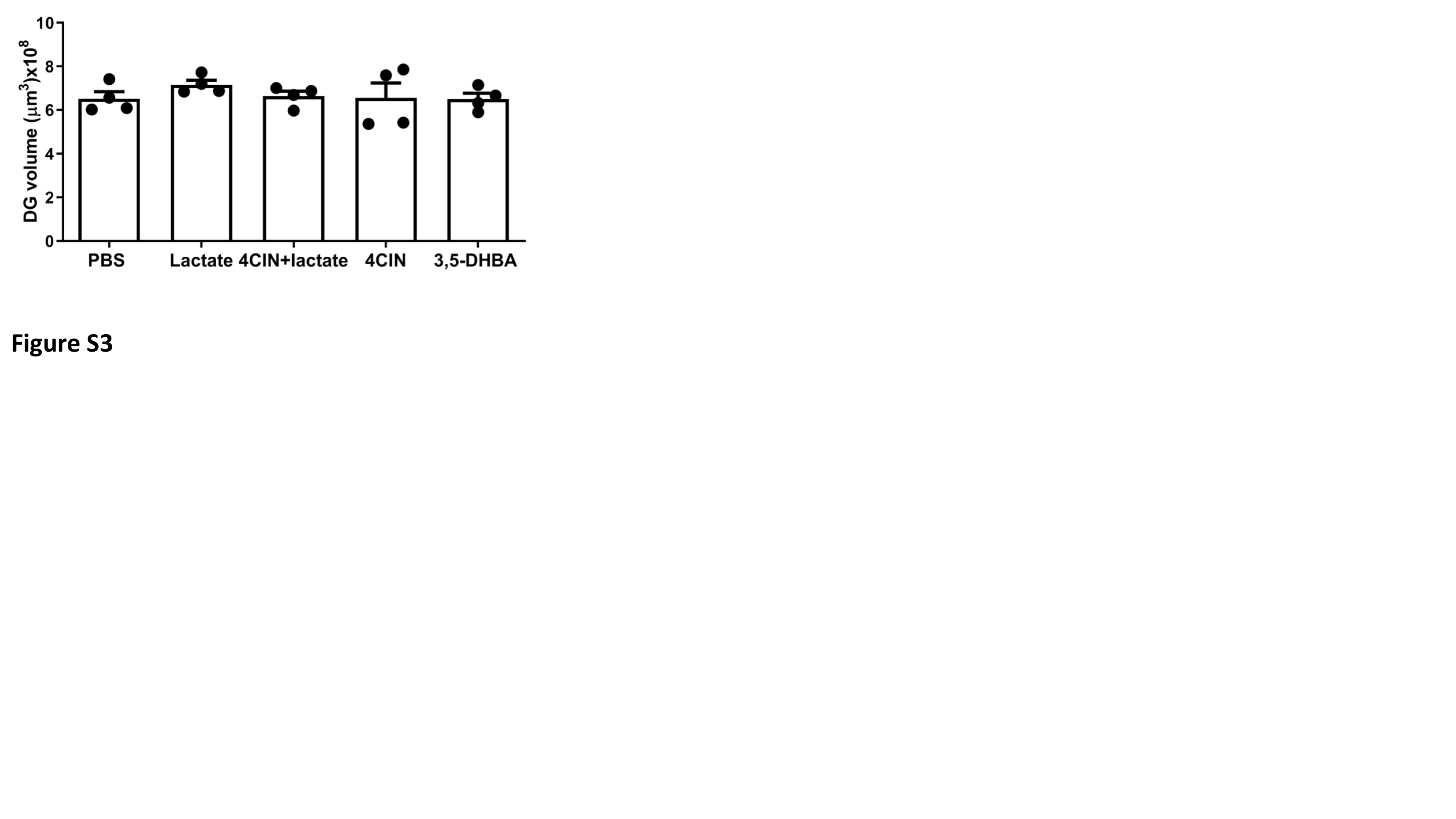

Supplement: Supplementary file 4 [file Image_3.TIFF]

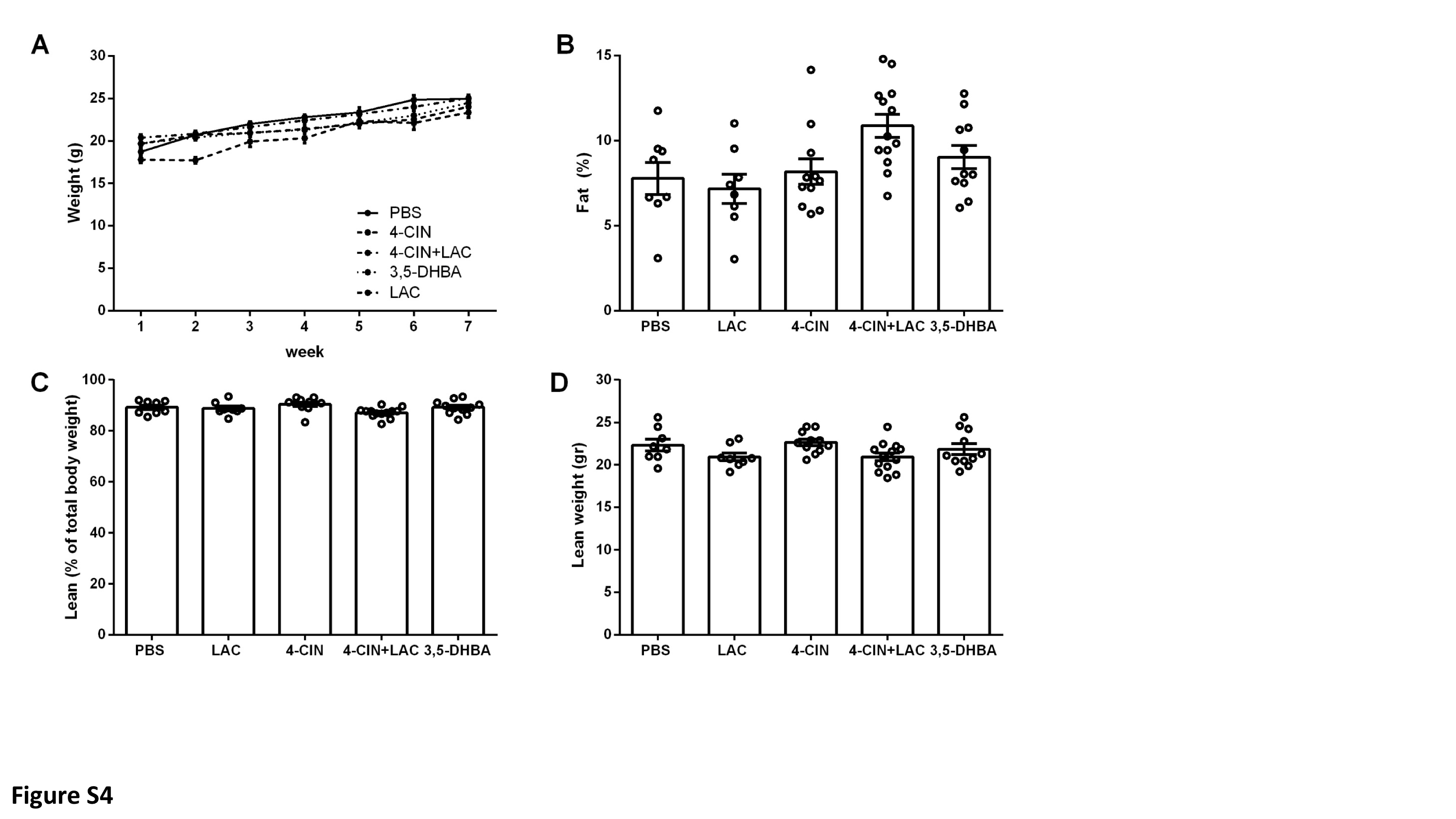

Supplement: Supplementary file 5 [file Image_4.TIFF]

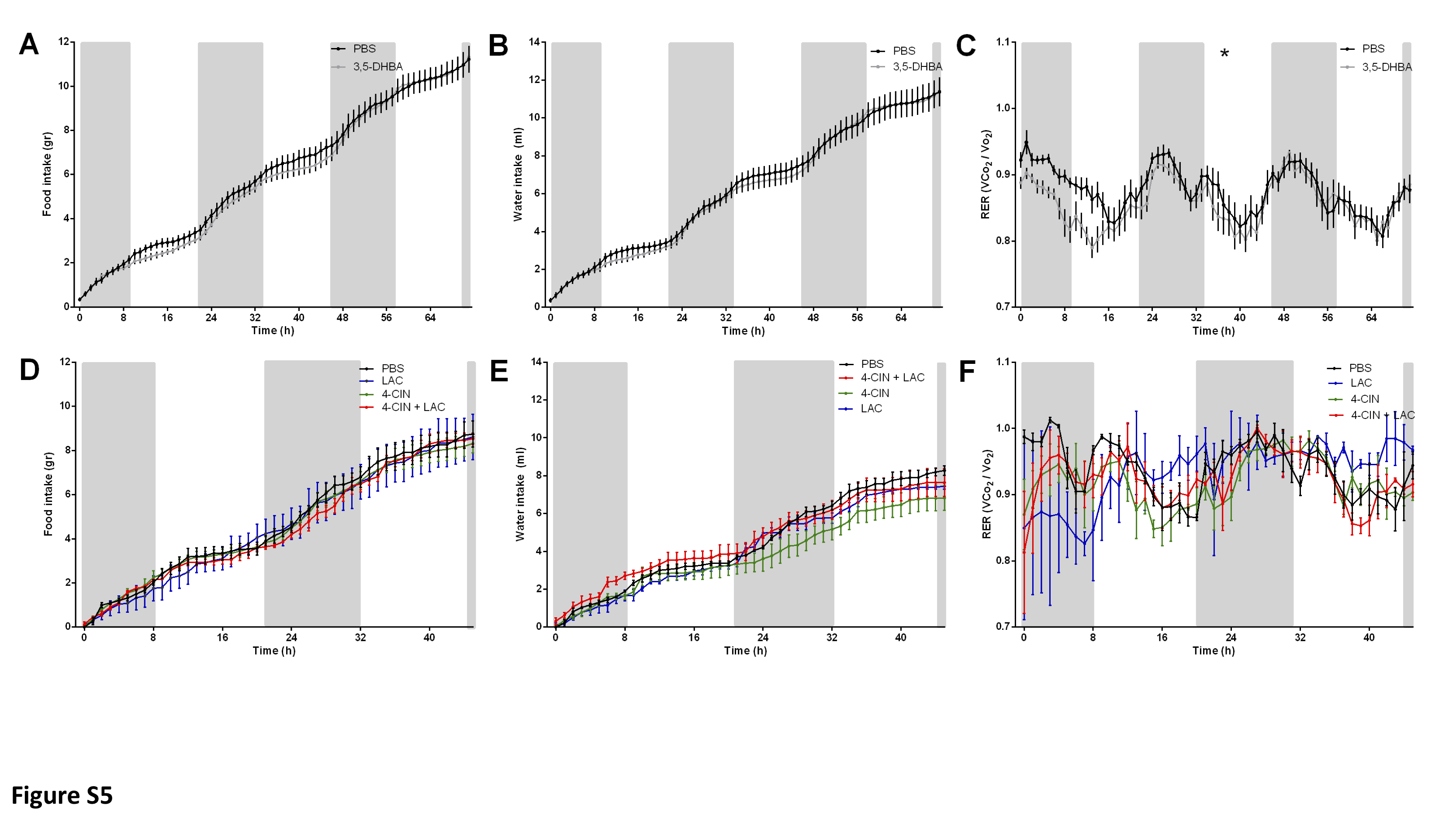

Supplement: Supplementary file 6 [file Image_5.TIFF]

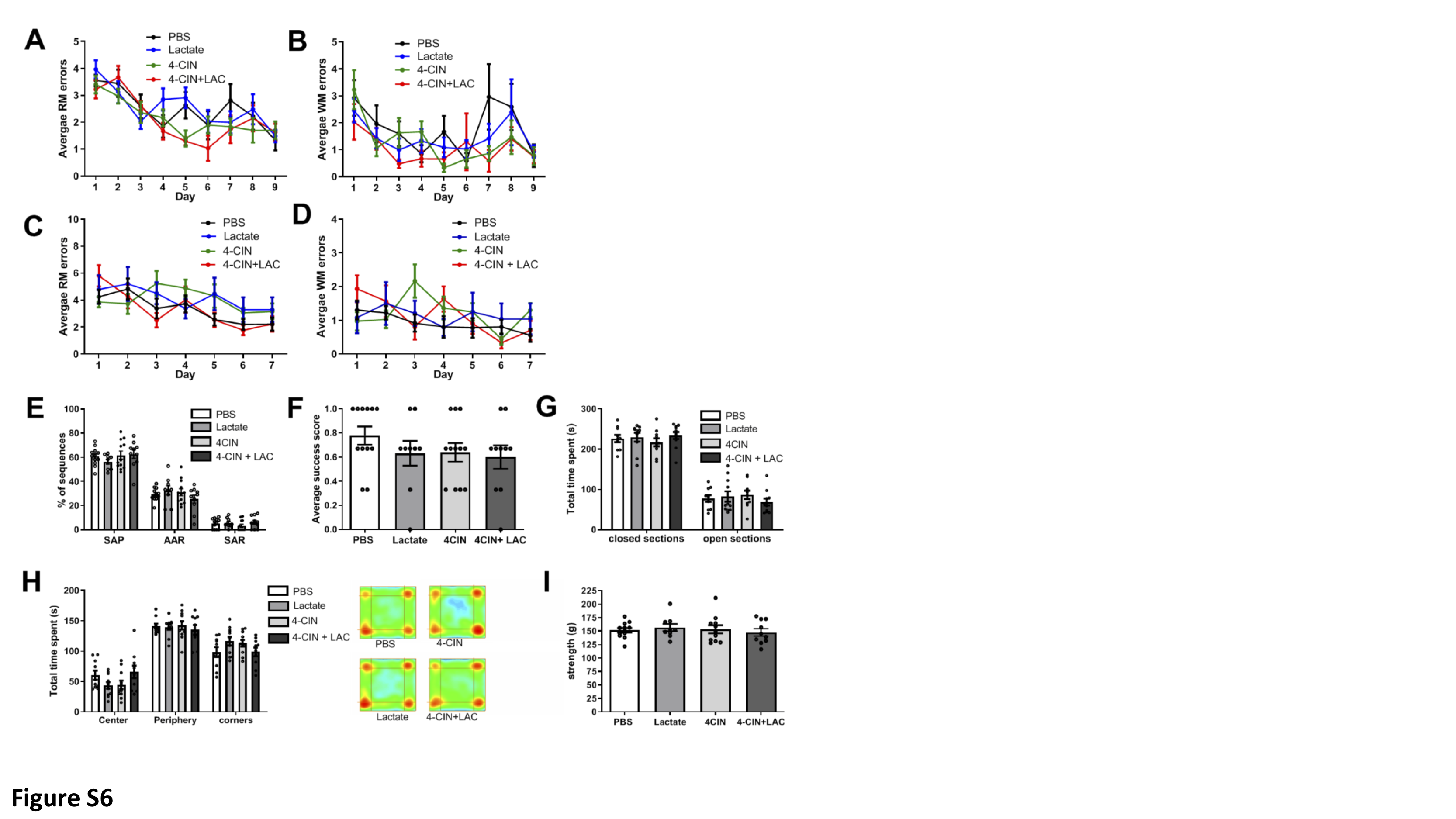

Supplement: Supplementary file 7 [file Image_6.TIFF]
